# Muscarinic acetylcholine activity modulates cortical silent period, but not motor evoked potentials, during muscle contractions

**DOI:** 10.1007/s00221-023-06616-7

**Published:** 2023-04-27

**Authors:** Lisa M. Dempsey, Justin J. Kavanagh

**Affiliations:** 1 Menzies Health Institute Queensland, Griffith University, Southport, Australia; 2 School of Allied Health Sciences, Griffith University, Gold Coast Campus, Southport, QLD 4222 Australia

**Keywords:** Acetylcholine, Neuromodulation, Promethazine, Muscle activation, Fatigue

## Abstract

This study used transcranial magnetic stimulation (TMS) to determine if muscarinic receptor blockade affects muscle responses during voluntary contractions. Motor evoked potentials (MEPs) were recorded from biceps brachii in 10 subjects (age: 23 ± 2) during 10%, 25%, 50%, 75%, and 100% maximal voluntary contractions (MVCs). Each contraction intensity was examined under non-fatigued and fatigued conditions. All measurements were obtained post-ingestion of 25 mg promethazine or placebo. MEP area and the duration of the TMS-evoked silent period (SP) were calculated for all contractions. No drug-related differences were detected for MEP area during non-fatigued or fatigued contractions. A main effect of drug was detected for the SP (*p* = 0.019) where promethazine increased SP duration by an average of 0.023 $\pm$ 0.015 s. This drug effect was only identified for the unfatigued contractions and not following the sustained fatiguing contractions (*p* = 0.105). The cholinergic system does not influence corticospinal excitability during voluntary muscle contractions, but instead affects neural circuits associated with the TMS-evoked SP. Given the prevalence of cholinergic properties in prescription and over-the-counter medications, the current study enhances our understanding of mechanisms that may contribute to motor side-effects.

## Introduction

Four primary neuromodulator systems exist in the central nervous system (CNS): dopaminergic, adrenergic, serotonergic, and cholinergic. While there is a good understanding of how the first three systems regulate the gain of motor circuits in the CNS, our understanding of how the cholinergic system regulates the excitability of motor pathways during voluntary muscle contraction is limited. Muscarinic acetylcholine receptors (mAChRs) are the predominant cholinergic receptor in the CNS, where activation of mAChRs initiates G-protein–coupled signal transduction pathways (Caulfield 1993; Felder 1995). In general, activation of mAChRs can cause a broad range of actions where inhibition, excitation, or combinations of both, can occur presynaptically or postsynaptically. Although this diversity in neural responses creates a unique challenge when examining muscle activation, animal preparations involving the hippocampus and pyramidal cells have provided great insight to mAChR mechanisms. For example, activation of the receptors has been shown to depolarize CA1 pyramidal neurons by inhibiting the resting potassium channels (Benardo and Prince 1982; Madison et al. 1987), and induce transient increases in Ca^2+^ concentration in CA1 astrocytes to signal the release of glutamate (Araque et al. 2002). More specific to motor pathways, activation of receptors inhibits afferents the regulate motoneurons by reducing glutamatergic activity via the inhibition of Ca^2+^ channels (Araque et al. 2001). Given that presynaptic mAChRs are largely autoreceptors (Dudar and Szerb 1969; Kilbinger et al. 1993), and post-synaptic mAChRs can be either inhibitory or excitatory (Brown 2010; Kuba and Koketsu 1978), it is difficult to predict how cholinergic activity affects motor activity based on individual receptor activity from cellular or animal preparations.

The use of atropine for the insight of anti-muscarinic effects within human studies has been vastly used, however, promethazine allows the same insight without as considerable side-effects. Promethazine is one of the most potent antihistaminergic drugs with obvious antimuscarinic and sedative effects (Cantisani et al. 2013). In the initial discovery and refining of antihistaminergic drugs, it was identified that the sedative effect of histamine was associated with the brain, however, not until 1987 were the main pathways demonstrated in a region known as the tuberomammillary nucleus (TMN) (Haas et al. 2008). The histamine neurons have been shown to project to noradrenergic nucleus locus coeruleus, the cholinergic nuclei of the brainstem, and the dopamine neurons, all structures associated with alertness (Samuels and Szabadi 2008). Further, promethazine proves to present a broad pharmacological activity in that while the noted antihistaminergic effects are apparent, there are significant atropine (anticholinergic) like affects (Halpern and Hamburger 1948). Further to this researchers have been moving towards using promethazine as a more accessible drug for organophosphorus compound poisoning (OPC) in replacement of atropine (Nurulain et al. 2015).

Transcranial magnetic stimulation (TMS) is a non-invasive tool that can be used to assess the excitability of cortical circuits and motor pathways in humans. A single TMS pulse applied to the motor representation of a muscle induces a motor evoked potential (MEP) in the target muscle. The amplitude of the MEP reflects the excitability of the corticospinal pathway, where facilitation of MEP responses progressively increases until 50–70% MVC for upper limb muscles (Taylor et al. 1997, 1996). With respect to mAChRs, blockade of these receptors with drugs such as scopolamine can facilitate larger MEPs in the resting first dorsal interosseus (FDI) and during FDI contractions of 20% MVC (Di Lazzaro et al. 2000). However, it is unknown if antimuscarinic-based increases in MEP are consistent across a wider range of contractions. Antimuscarinic drugs have also been used to demonstrate that cholinergic effects reduce GABA_A_ mediated short-latency-inhibition with a constant level of voluntary contraction and reduce intracortical inhibition at rest (Di Lazzaro et al. 2000; Liepert et al. 2001). This possibly suggests that other inhibitory GABAergic circuits, such as those involved in the TMS-evoked silent period, may also be affected by anticholinergic drugs. The initial 50–80 ms of the SP is thought to be due to spinal mechanisms (Cantello et al. 1992), whereas the latter component of the SP is thought to reflect gamma-aminobutyric acid (GABA) intraneuronal activity that arises from GABA_B_-mediated intracortical inhibition (McDonnell et al. 2006). Thus, if antimuscarinic drugs enhance activity in inhibitory circuits associated with the motor pathway, it would likely be reflected in an overall lengthening of the TMS-evoked SP in humans.

The purpose of this study was to assess if muscarinic receptor blockade modulates TMS-evoked MEPs and SPs during voluntary muscle contractions. The target muscle in this study was the biceps brachii, where EMG responses to single TMS pulses were recorded during elbow flexions of 10%, 25%, 50%, 75%, and 100% of MVC. To determine if muscarinic receptors have a neuromodulatory role when the motor system is fatigued, the range of contraction intensities were again examined. However, this time they were preceded by a sustained maximal effort elbow flexion that reduced the individuals force generating capacity to 60% of their initial MVC. It was hypothesised that a mAChR blockade would increase biceps brachii MEP area, and lengthen biceps brachii SP, during the voluntary elbow flexions regardless of whether muscle fatigue was present. Moreover, it was hypothesized that these antimuscarinic effects would be most prominent during higher contraction intensities when voluntary drive from the motor cortex to the target muscle is high.

## Methods

### Participants

Ten healthy individuals (mean age 23 ± 2) were involved in the study. Each participant attended two sessions spaced 10 days apart. Participants were screened using a modified medical history questionnaire that identified contraindications to promethazine, TMS, percutaneous electrical stimulation, and the exercise tasks employed in this study. No participant was taking any form of CNS medication that could impact measurements.

### Compliance with ethical standards

Written and informed consent was obtained before the commencement of testing. This experiment was approved by the Griffith University Human Research Ethics Committee and conformed to the standards set by the Declaration of Helsinki.

### Experiment design and drug administration

This study was a human, double-blinded, placebo-controlled, crossover design. Participants were administered either a placebo or 25 mg promethazine capsule. Promethazine was chosen for its high potency and antagonistic affects upon mAChRs (Kubo et al. 1987). The administration of capsules was randomised to avoid order effects. Two hours after administration of the capsule experimental testing occurred. This timing was chosen to coincide with reported peak plasma concentration of promethazine, which typically occurs between 1.5 and 3 h (Taylor et al. 1983).

### Force and EMG recording

**Fig. 1:**
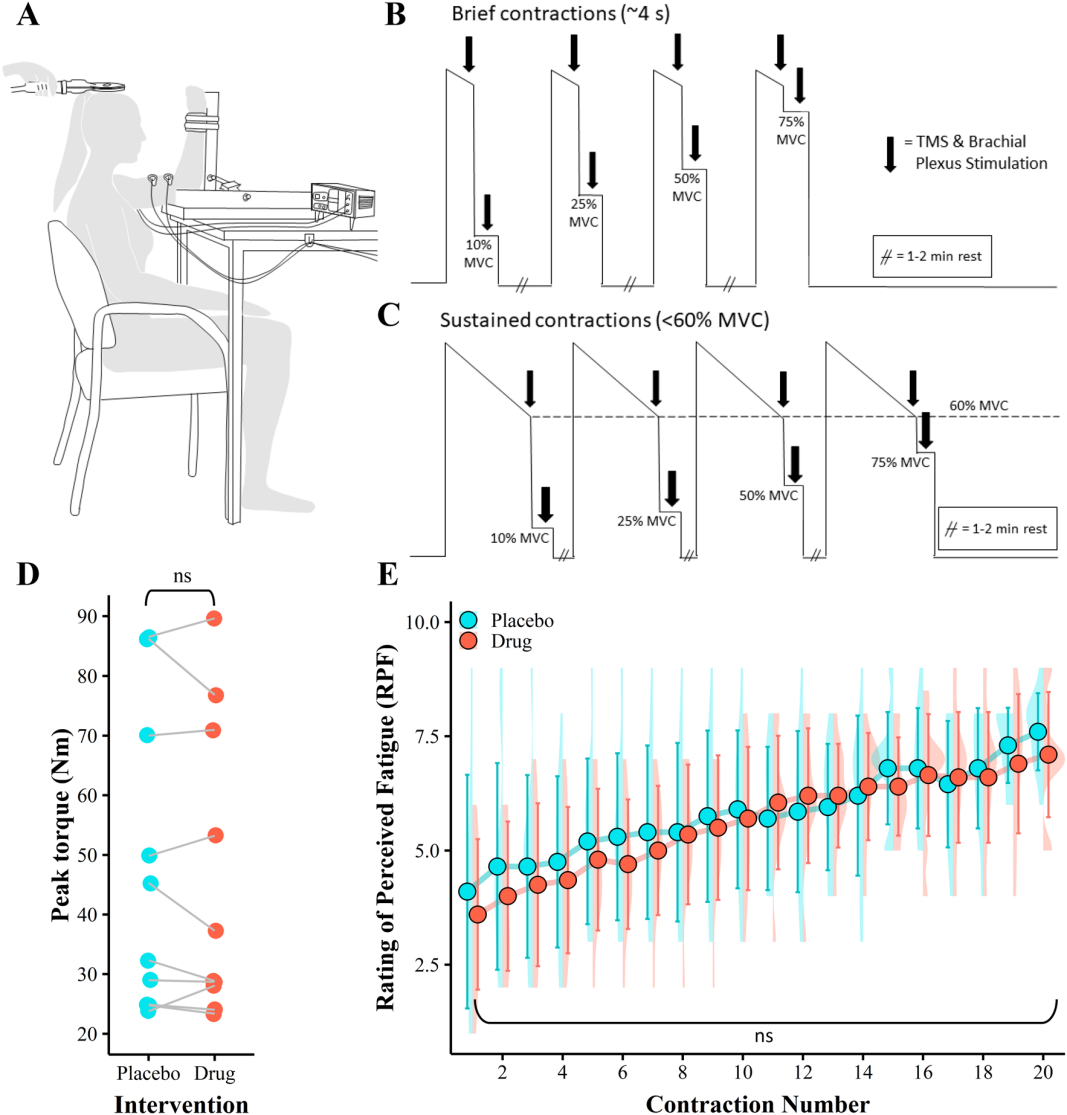
Participants were seated with their arm attached to a custom designed force transducer. Biceps brachii EMG was recorded from the right limb following the administration of promethazine or a placebo (**A**). Two contraction protocols were implemented. Protocol 1 consisted of a brief maximal voluntary contraction (MVC) followed by brief (4 s) submaximal contraction of 10%, 25%, 50%, or 75% MVC with 1–2 min rest between each contraction (**B**). Protocol 2 consisted of a sustained MVC followed by brief submaximal contractions (10%, 25%, 50%, 75% MVC) with 1–2 min between each contraction (**C**). Cortical and brachial plexus stimulations were delivered once sustained MVC force declined to 60% of the initial unfatigued MVC. Submaximal contractions in protocol 2 were based off the (fatigued) 60% MVC. Cortical and Brachial plexus stimulation again occurred during brief submaximal contractions once participant had met force target. Unfatigued peak torque data for each participant was recorded prior to protocols, no significant effect of intervention was detected (*p* = 0.449, **D**). Ratings of perceived fatigue (RPF) were taken throughout protocol 2, no significant effect of intervention was detected (*p* = 0.700, **E**). Torque and RPF data are presented as the means $\pm$ 1 standard deviation of the mean. Data distribution is represented with split violin plots, *ns* non-significant

Participants were seated with their elbow flexed and held at 90° in a custom designed force transducer (Fig. 1A). An S-Beam load cell (PT4000, 1.1 kN range, full scale output 3 mV/V) capable of measuring both compression and tension were used to measure isometric elbow flexion. To calculate torque (Nm), the force (N) generated by each participant was multiplied by the distance between their lateral epicondyle and the wrist strap which attached them to the transducer. Torque data were sampled at 2000 Hz using a 16-bit CED Power 1401 data acquisition interface and Spike2 v7.02a software (Cambridge Electronic Design). Surface EMG was recorded from surface electrodes affixed to the biceps brachii in a bipolar arrangement (24 mm Kendall Arbo electrodes, 40 mm interelectrode distance). EMG signals were amplified (× 300), filtered (20–2000 Hz) and sampled (2000 Hz) using a CED1902 amplifier (Cambridge Electronic Design).

### Motor cortical stimulation

A circular coil with a 90 mm outside diameter was positioned over the vertex of the head and delivered single-pulse stimulations to the motor cortex via a MagStim 200^2^ TMS unit. The direction of flow in the coil preferentially activated the left motor cortex (anterior–posterior current). Given that voluntary and sustained voluntary contractions can cause alterations in action potential propagation at the level of the muscle, we normalised MEPs to the maximal direct motor response of the muscle (Mmax) (Carroll et al. 2017; Martin et al. 2006). Mmax was obtained via single supramaximal stimuli (100 $\mu s$ duration) delivered to the brachial plexus 2 s following each TMS pulse. A constant-current stimulator (DS7AH, Digitimer) was used for the electrical stimulation with the cathode placed over the supraclavicular fossa (Erb’s point) and anode placed over the acromion. After producing a resting Mmax, the stimulus intensity was set to 30% above the Mmax for experimental testing (91–195 mA).

### Active motor threshold (AMT)

Each participant’s motor threshold was determined during light contraction of the biceps brachii. To ensure consistency between participants and between sessions, a horizontal cursor of 0.01% peak-to-peak Mmax was displayed on a monitor, and participants were required to track the cursor with their rectified biceps brachii EMG amplitude (0.2 s bins). TMS intensity was adjusted by 1% MSO increments in that at least five out of ten TMS pulses elicited an MEP greater than 100 V$\mu$ (Rossini et al. 1994, 2015). The TMS intensity corresponding to this criterion was defined as the AMT.

### TMS stimulus–response curves

Muscle responses were obtained from the lightly contracted biceps brachii from 90% AMT to 190% AMT in 10% AMT increments in a semi-randomised protocol. Six stimulations were delivered for each of the 11 stimulator intensities, where a 20 s rest was provided between each stimulation. One participant did not reach 190% AMT as the intensity would have been higher than 100% MSO. MEP area was subsequently normalised to Mmax. The TMS intensity that produced the largest normalised MEP with the smallest stimulator output was chosen as the TMS intensity for the remainder of the testing session (54–84% MSO).

### Contraction protocol 1: unfatigued muscle

Participants performed 5 maximal effort contractions, where the trial that produced the largest torque was deemed the participants maximal voluntary contraction (MVC). Cortical and brachial plexus stimulation was delivered during initial baseline contractions, with a minimum of 2 min rest occurring between each effort. Target torques of 10%, 25%, 50%, and 75% MVC were then calculated from the measured MVC and displayed on a monitor. Participants performed 5 contractions for each target torque, whereby a maximal contraction was followed immediately by a submaximal contraction with no relaxation of the muscles when reducing force to submaximal contraction. Each maximal contraction within protocol 1 was a brief (~ 4 s) maximal contraction followed directly by a brief (4 s) submaximal contraction (Fig. 1B). Cortical and brachial plexus stimulation was delivered during both the maximal and submaximal contraction. A rest period of 1–2 min occurred between contractions, where each contraction intensity was semi-randomised.

### Contraction protocol 2: fatigued muscle

After the unfatigued protocol was completed, a second protocol was performed that involved a fatigue-inducing sustained maximal contraction. Each participant held a maximal contraction until their force had reduced to 60% of their initial unfatigued MVC. This ensured that each trial, and each participant, had the same level of fatigue (decline in force) for neurophysiological assessments. Immediately after 60% MVC was reached, the participant performed a brief (4 s) submaximal contraction (10%, 25%, 50%, or 75% MVC). The submaximal contractions were based directly off the (fatigued) 60% MVC. Participants performed 5 contractions for each target torque. Each maximal contraction within protocol 2 was a sustained maximal contraction, in which the cortical and brachial plexus stimulation were delivered once the participants force had dropped to 60% of initial MVC. The sustained maximal contractions were followed directly by a brief (4 s) submaximal contraction (Fig. 1C) with no relaxation before the reduction in force. Cortical and brachial plexus stimulation was delivered once the participant has obtained the submaximal target force. A rest period of 1–2 min occurred between contractions.

#### Data analysis

Data were analysed using built-in functions in Spike2 v7.02a software (Cambridge Electronic Design). MEP area and Mmax area were calculated from biceps brachii EMG following cortical and brachial plexus stimulations, respectively. MEP area and Mmax area was calculated from the first deflection from baseline created by the stimulation to the end of the waveform (which was defined as the return to baseline after all phases of the wave had ended). MEPs were normalised to Mmax, and then averaged for each contraction intensity. TMS-evoked silent period duration was calculated as the time from the stimulus artifact to the return of EMG activity following the TMS pulse (SP was calculated in Spike2 by placing cursors at each event). SPs were averaged for each contraction intensity.

#### Statistical analysis

**Fig. 2:**
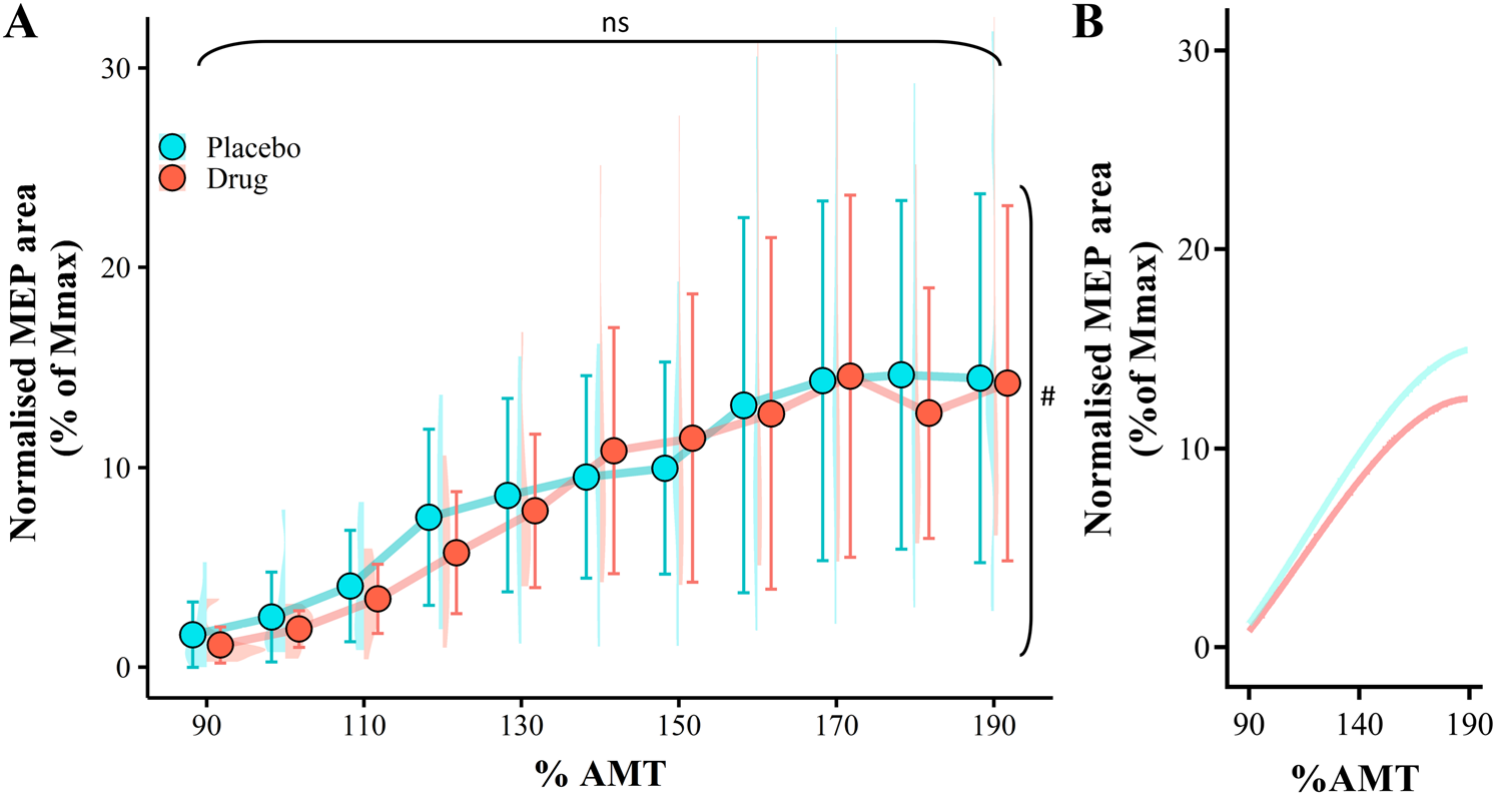
Input–output curve for MEP area (**A**). Data are presented for 90% active motor threshold (AMT) through 190% AMT for lightly active biceps brachii muscle. Curve fitting was performed by modelling the MEP data using third-order polynomial equations (**B**). All data were normalised to individuals Mmax. MEP data are presented as the means $\pm$ 1 standard deviation of the mean. Data distribution is represented with split violin plots, ns = non-significant main effect of drug), ^#^significant main effect of contraction intensity (**A**. *p* < 0.001)

All statistical analyses were performed in R, using RStudio (version 2022.02.0 + 443 "Prairie Trillium" release, Boston, MA) where significance level of *p* < 0.05 was set for all tests. Data for the TMS stimulus–response curves were entered into a linear mixed effects growth model where a two-way mixed ANOVA was used to examine the main effects of drug (promethazine vs placebo) and stimulation (90% AMT to 190% AMT in 10% increments) on normalised MEP area. The growth model was fit to account for participant ID for all comparisons. The data were then also modelled to generate a stimulus–response curve, each participant was considered an independent replicate. Thus, individual MEPs at a given stimulation intensity were the average of 10 TMS pulses. A series of equations were fit to the data, including Boltzman sigmoidal, First, Second and Third order polynomials. The best fit was deemed by the highest coefficient of determination (${R}^{2}$) value. A third order polynomial equation best modelled the data for the TMS stimulus–response (placebo: ${R}^{2}$ = 0.57, sum of squares = 44.14; promethazine: ${R}^{2}$ = 0.73, sum of squares = 23.49, Fig. 2B). Paired sample *t*-tests were used to assess the effect of drug on AMT. Torque data were entered into a one-way ANOVA to assess the main effect of drug on torque output. Shapiro–Wilk's test of normality was applied to the *T*-tests. For protocol 1 and protocol 2 data were entered into a linear mixed effects model. Separate repeated measures two-way ANOVAs were used to examine the main effect of drug (promethazine vs. placebo) and contraction intensity (10%, 25%, 50%, 75% and 100% MVC) on MEP area and silent period duration. Shapiro–Wilk's test of normality was applied to all two-way repeated measures ANOVA, with Mauchly's test of sphericity. Greenhouse–Geisser corrections were used if sphericity was violated for any test. In the event of a significant interaction effect or main effect of stimulation, post hoc analysis was performed with Bonferroni adjustment.

## Results

### Active motor threshold and TMS stimulus–response

Promethazine did not affect AMT as there were no drug-related differences in stimulator output when MEPs were first detected in the lightly contracting muscle (*t*(9) = 0.520, *p* = 0.616, placebo = 43.0 $\pm$ 6.0% MSO, promethazine = 41.8 $\pm$ 6.3% MSO, Fig. 2A). Similarly, promethazine did not affect the TMS stimulus–response during light muscle contraction. Although there was a main effect of stimulation intensity (*F*(1,206) = 25.514, *p* < 0.001), where an increase in stimulator output corresponded to an increase in MEP area, there was no main effect of drug detected in MEP area for the stimulus–response curve (*F*(1, 206) = 0.024, *p* = 0.876, Fig. 2). There was no drug by stimulator intensity interaction effect detected for MEP area (*F*(1, 206) = 0.597, *p* = 0.441).

### Maximal voluntary contraction torque

Promethazine did not affect maximal elbow flexion torque as there were no drug-related differences in MVC (*F*(1,9) = 0.63, *p* = 0.499, Fig. 1D). The amplitude of muscle activity was not affected by promethazine during MVC as there were no drug-related differences in EMGrms during the MVC (*F*(1,7) 1.91, *p* = 0.210. Promethazine did not affect perceived fatigue levels as there were no drug-related differences in RPF (*F*(1,9) = 0.16, *p* = 0.700, Fig. 1E).

### MEP and silent period duration during brief voluntary contractions.

**Fig. 3:**
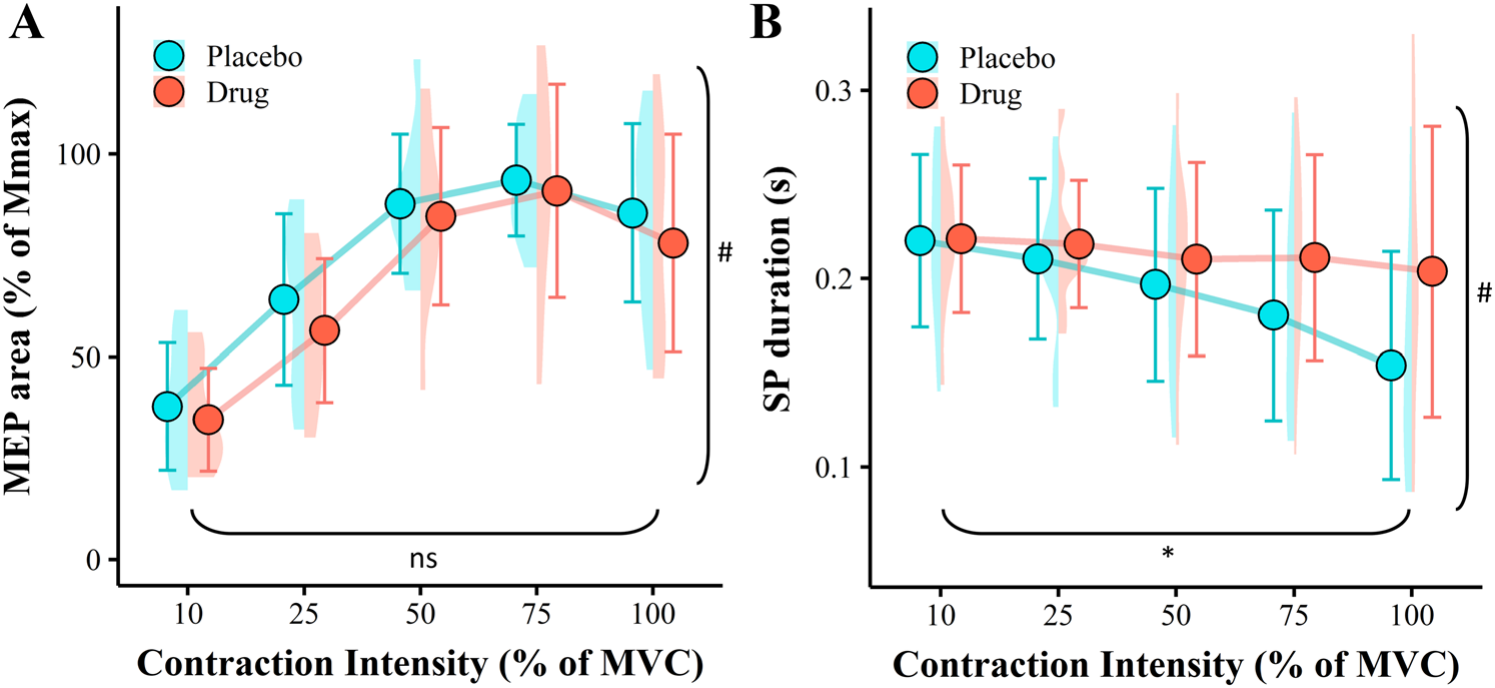
Protocol 1 MEP area (**A**), and TMS-evoked silent period (**B**). Data are biceps brachii muscle during brief maximal and submaximal voluntary contractions (10%, 25%, 50%, and 75% MVC). Data are normalised to individuals Mmax at corresponding voluntary contraction level. Data are presented as the means $\pm$ 1 standard deviation of the mean. Data distribution is represented with split violin plots, *ns* non-significant main effect of drug, *significant main effect of drug (*B*. *p* = 0.019), ^#^significant main effect of contraction intensity (**A**. *p* < 0.001, **B**. *p* < 0.001)

There was no main effect of drug on MEP area during brief unfatigued voluntary contractions (*F*(1, 9) = 0.77, *p* = 0.402, Fig. 3A). However, there was a main effect of contraction intensity (*F*(1.70, 15.33) = 33.06, *p* < 0.001, where MEP area progressively increased from 10% MVC (36.2 ± 9.4 mV) through to 75% MVC (92.2 ± 11.5 mV) before reducing slightly when performing a 100% MVC (81.8 ± 14.3 mV). There was no drug by contraction intensity interaction effect for MEPs during brief voluntary contractions (*F*(2.39, 21.48) = 0.30, *p* = 0.780).

There was a main effect of drug identified for the TMS-evoked SP (*F*(1, 9) = 8.06, *p* = 0.019, Fig. 3B), where SP duration was greater for the promethazine condition compared to the placebo condition (average increase: 0.023 $\pm$ 0.015 s). There was also a main effect of contraction intensity, where SP duration decreased from 10% MVC (0.221 $\pm$ 0.012 s) to 100% MVC (0.179 $\pm$ 0.019 s, *F*(2.92, 26.25) = 6.97, *p* < 0.001). No drug by contraction intensity interaction effect was detected for SP duration (*F*(1.70, 15.34) = 3.41, *p* = 0.066).

### MEP and silent period duration following fatiguing sustained contractions

**Fig. 4:**
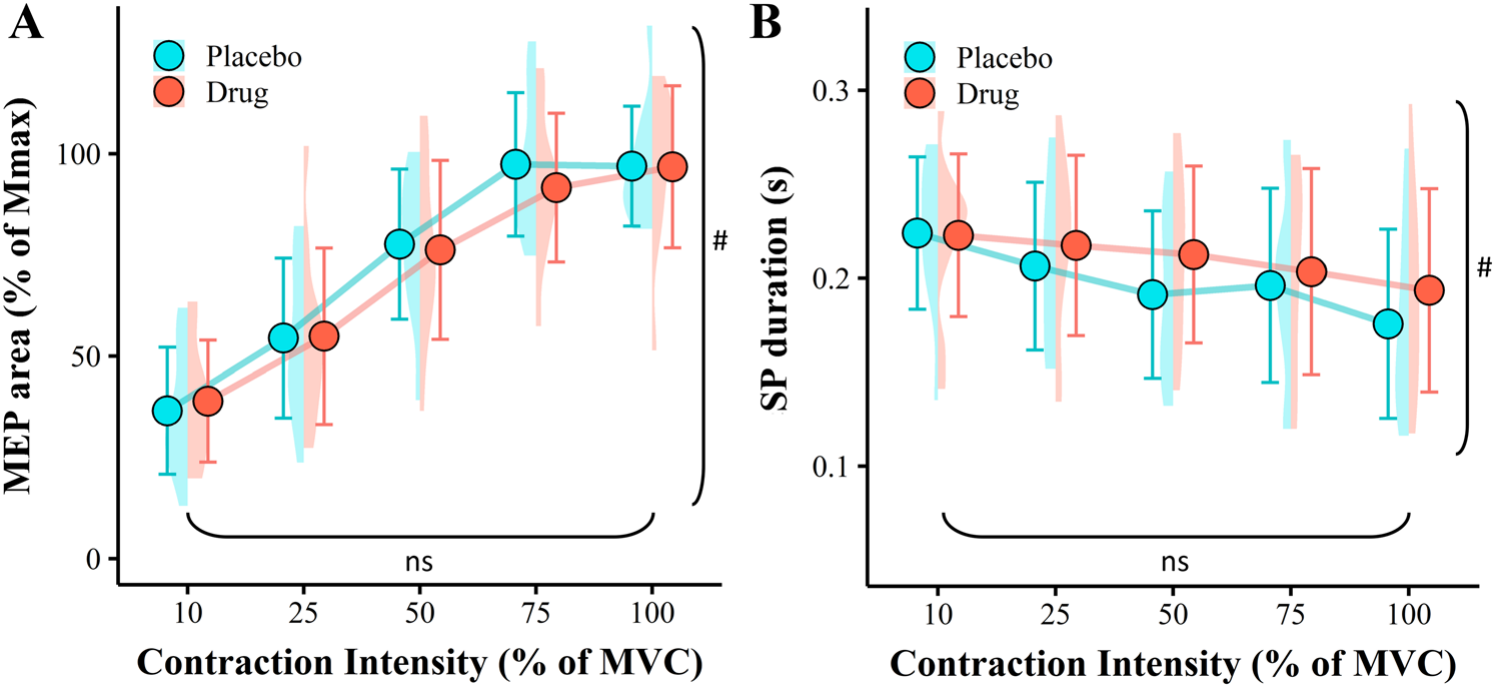
Protocol 2 MEP area (**A**), and TMS-evoked silent period (**B**). Data are biceps brachii muscle during sustained maximal and brief submaximal voluntary contractions (10%, 25%, 50%, and 75% MVC). Data are normalised to individuals Mmax at corresponding voluntary contraction level. Data are presented as the means $\pm$ 1 standard deviation of the mean. Data distribution is represented with split violin plots, *ns* non-significant main effect of drug, ^#^significant main effect of contraction intensity (**A**. *p* > 0.001, **B**. *p* > 0.001)

There was no main effect of drug on MEP area following the maximal effort sustained contractions (*F*(1, 9) = 0.06, *p* = 0.818, Fig. 4A). However, similar to the unfatigued contractions, a main effect of contraction intensity was detected where MEPs increased from 10% MVC (37.7 $\pm$ 10.6) through to 100% MVC (96.9 $\pm$ 9.8, *F*(1.82, 16.42) = 43.35, *p* > 0.001). The was no drug by contraction intensity interaction detected for MEP area following the sustained voluntary contractions (*F*(2.46, 22.11) = 0.35, *p* = 0.751).

Unlike the unfatigued contractions, there was no main effect of the drug detected for TMS-evoked SP duration following the sustained voluntary contractions (*F*(1, 9) = 3.25, *p* = 0.105, Fig. 4B). However, there was a main effect of contraction intensity, where SP duration decreased from 10% MVC (0.223 $\pm$ 0.0129 s) to 100% MVC (0.185 $\pm$ 0.0159 s, *F*(3.29, 29.59) = 10.18, *p* > 0.001). No drug by contraction intensity interaction effect was identified for SP duration (*F*(2.74, 24.67) = 2.44, *p* = 0.093).

## Discussion

The present study assessed the effects of a potent antimuscarinic drug on MEP area and TMS-evoked SP during voluntary contractions. To achieve this, a contraction protocol was used to assess cholinergic effects during brief contractions of 10%, 25%, 50%, 75%, and 100% MVC. This was followed by a second protocol that assessed MEPs during the same contraction intensities, however, a consistent within-subject and between-subject level of fatigue was induced before each contraction. The main finding was that the antimuscarinic drug reduced the TMS-evoked SP during unfatigued contractions but did not affect MEPs under any contraction condition. This suggests that the cholinergic system does not influence corticospinal excitability during voluntary muscle contractions, but instead affects inhibitory circuits associated with the TMS-evoked silent period.

### Silent period is affected by antimuscarinic activity during unfatigued voluntary contractions

The current study revealed that SP duration shortened during voluntary muscle contractions, where the duration of TMS-evoked SP progressively reduced with an increase in contraction intensity. The relationship between SP and contraction intensity has been reported on a number of occasions, where many studies report that SP duration is unaffected by voluntary contractions (Haug et al. 1992; Inghilleri et al. 1993; Kojima et al. 2013; Saisanen et al. 2008; Taylor et al. 1997; Wu et al. 2002), and only a few report that a reduction in SP duration occurs with muscle contractions (Cantello et al. 1992; Hammond and Vallence 2007; Wilson et al. 1993). A large emphasis has been put on the instruction to the participant while performing the contraction task, Mathis et al. (Mathis et al. 1998) have shown this from instruction to perform an additional voluntary contraction of the biceps brachii muscle “immediately after” the TMS pulse. This instruction resulted in shorter SP durations compared to maintaining a constant force level. This concept was made obvious within piloting data with the experimenter giving precise and consistent instruction to participants when completing the protocol. A possible factor that delineates the studies that report changes in SP is the orientation of the stimulating coil, where an anterior–posterior current may have greater efficiency for activating inhibitory cortical circuits compared to a posterior-anterior currents (Matsugi 2019). PA induced current in the brain evokes a single descending wave that is thought to originate from the activation of monosynaptic corticocortical connections projecting onto corticospinal neurones (Di Lazzaro et al. 2012). The use of a PA orientation has been related to the later volleys in a representation of I-waves, further most likely to be stimulating corticocortical axons which further project onto corticospinal neurons, or the axons of additional corticofugal systems with similar projections (Di Lazzaro et al. 2008). After establishing that our anterior–posterior current did, in fact, reduce SP duration in biceps brachii during voluntary contractions, the reduction in SP duration was ameliorated with mAChR blockade. Although it was hypothesized that cholinergic effects would be dependent on the level of neural drive from the motor cortex to the muscle, the antimuscarinic effects on SP were present regardless of contraction intensity.

The initial 50–80 ms of the TMS-evoked SP has been linked to spinal mechanisms such as recurrent inhibition, after-hyperpolarization of activated motoneurons, and activation of 1a inhibitory interneurons (Duchateau and Baudry 2014; Ozyurt et al. 2019). Given that mAChRs are present on spinal interneurons which provide synaptic input to motoneurons, we cannot rule out that antimuscarinic effects may have contributed to early spinal inhibition following cortical stimulation. However, we contend that the cholinergic activity in the motor cortex was the primary contributor to our SP results. Cholinergic projections from brainstem nuclei to the cortex are vast, are diffuse, and are known to promote regional plasticity in the hippocampus and neocortex (Auerbach and Segal 1996; Dennis et al. 2016). Moreover, TMS studies have revealed that selective cortical inhibition (such as short afferent inhibition and short-latency intracortical inhibition) is reduced with the antimuscarinic scopolamine and atropine in the absence of muscle contractions (Di Lazzaro et al. 2000; Liepert et al. 2001). Given that TMS studies also report that SP is increased with GABA_B_ receptor agonists (Siebner et al. 1998; Stetkarova and Kofler 2013), the findings in the current study reinforces the viewpoint that the cholinergic system innervates inhibitory GABAergic circuits in the motor cortex.

The lengthening of SP due to promethazine contrasts with two previous TMS studies exploring anticholinergic effects in the CNS. However, there are a number of factors that differ between previous studies and the current investigation. The study of Di Lazzaro (Di Lazzaro et al. 2000) did not find any change in first dorsal interosseus SP following the administration of intravenous scopolamine. These results were in a sample of three participants, and unlike the current study the SP was assessed in a distal muscle and only during the performance of a 20% MVC. Similarly, Liepert et al. (Liepert et al. 2001) examined first dorsal interosseus SP during the performance of a 30% MVC and did not find significant drug-effects following the oral administration of 2 mg atropine. However, it is interesting that atropine caused an average increase in SP of 20 ms 1 h after ingestion, which is a similar to our SP duration following promethazine ingestion. Presumably, the lower sample size of 7 in the atropine study compared to our sample size of 10 played a role in these non-significant findings.

### Antimuscarinic activity did not affect MEP area during unfatigued voluntary contractions

A hypothesis of the current study was that mAChRs would modulate MEPs. However, there were no antimuscarinic effects detected for MEP area measured during a full range of voluntary contraction intensities. While the TMS-evoked SP involved inhibitory processes that are mediated by GABA_B_ receptor activity, the magnitude of MEPs are governed by both voltage-gated ion channels in the motor system and neuromodulating neurotransmitters. The prevention of mAChR activity did not influence MEP area which suggests that (1) synaptic input from descending pathways was increased to compensate for a reduction in neuromodulation, or (2) mAChRs do not play a significant role in modulating corticospinal excitability during muscle contractions. Given that there were no drug-related differences in AMT, input–output curves, or maximal torque generation (which target contraction intensities were calculated from), it is unlikely that synaptic inputs to motoneurons were affected with promethazine. Instead, it appears that ACh may not have the same influence on regulating corticospinal excitability compared to neuromodulators such as dopamine, noradrenaline, and serotonin. We recently demonstrated that muscarinic receptor blockade increases MEP area immediately following a 10 s MVC and a 60 s MVC of a small hand muscle (Dempsey and Kavanagh 2021). However, it is worth noting that these effects were most obvious in the few seconds following the contraction. That is, after a few seconds of the recovery the MEPs were once again similar to baseline for both placebo and promethazine conditions. Given that the current study did not uncover drug-effects during strong voluntary contractions, and our previous work revealed that muscarinic blockade has very subtle effect on MEP only following strong contractions, there is mounting evidence to suggest that acetylcholine has a limited role in regulating the excitability of the corticospinal tract.

### The cholinergic system did not modulate MEPs or SPs under conditions of fatigue

Fatigue-inducing contractions cause a reduction in the maximal force generating capacity of the muscle, where the decline in force may be due to an inability of the nervous system to activate the muscle, or a failure of the muscle itself to contract. The current study normalised the amount of fatigue that was induced for each participant and assessed corticospinal excitability by normalising the MEP to Mmax. These procedures allow us to identify the role that mAChR blockade has on fatigue-related central motor responses. Surprisingly, the administration of promethazine did not affect any measurement of corticospinal excitability (or TMS-evoked SP), which suggests that mAChRs have no role in muscle activation while experiencing significant amounts of fatigue. Our previous work examined antimuscarinic effects on MEP area immediately following a fatigue-inducing 60 s MVC. In doing, we revealed that MEP area was increased from resting levels by 153% for a promethazine condition and 131% for a placebo condition, with MEPs for both conditions returning to resting levels in less than 10 s (Dempsey and Kavanagh 2021). The current study builds upon this work by clarifying that cholinergic changes in MEP were an after-effect of prolonged muscle activation. Moreover, our current data indicate that mAChR activity does not play a role in regulating corticospinal excitability during fatigued voluntary contractions.

### Considerations

Although we have attributed our findings for the motor system to anticholinergic effects, we must acknowledge that the medication used in this study also has strong antihistaminergic effects. We have previously used single choice, and multiple choice, reaction time paradigms to demonstrate that CNS acting antihistamines can influence the ability to react quickly (Kavanagh et al. 2012). However, our results pointed more towards movement effects being attributed to M receptor activity rather than H receptor activity. In particular, we have identified reaction time deficits following the administration of antihistamines that are not designed to cross the blood–brain barrier and therefore cannot target cortical-based H receptors. It is important to note that antihistaminergic effects are most likely to affect cognition and the decision-making process (such as reaction time paradigms) due to sedation or drowsiness (Hindmarch et al. 2001). Thus, it is unlikely that the histaminergic system played a large role in our findings, as the participants were only required to perform a steady-state contraction while the examiner activated the motor system via TMS (i.e. no cognitive challenge or decision making). Further, the histaminergic projections from the TMN have been shown to make a generalised neural activation to the maintenance of arousal (Moruzzi and Magoun 1949; Olson and Fuxe 1971; Panula et al. 1991). It has recently been revealed that the central acting components of histamine have motor cortical projections (Peng et al. 2023), thus, the central motor response cannot fully be reduced to mAChRs from the current study. Another potential explanation for this result includes the ceiling effect that the pain from the neuromuscular fatigue may have upon the MEP and motor output. A study using an intramuscular injection of hypertonic saline into the vastus lateralis, saw that muscle pain experienced decreases the contraction performance from the neuromuscular fatigue that is central in origin (Norbury et al. 2022). Following the sustained contraction neuromuscular fatigue may have caused participants to firstly perform at a reduced performance, while also resulting in a ceiling effect on neurophysiology measures. These ceiling effects have been a consideration for several studies (Poston et al. 2012), however, has recently been shown that further increases were seen within SP measures following fatigue (Brownstein et al. 2020). This further suggests that even in the presence of high stimulus intensities there is not an elicit ceiling effects in SP duration. Several other studies that have utilised the same method surrounding stimulus intensity found that MEP responses were still increased with fatigue (Aboodarda et al. 2020; Kennedy et al. 2016; Pageaux et al. 2015).

## Conclusions

While there is a good understanding of how the neuromodulators dopamine, adrenaline, and serotonin regulate the gain of motor circuits in the CNS, our understanding of how the cholinergic system regulates the excitability of motor pathways during voluntary muscle contraction is limited. The current study provides new insight to how the cholinergic system, and more specifically mAChRs, contribute to MEPs and TMS-evoked-silent period during muscle contractions. Our results indicate that the cholinergic system does not influence corticospinal excitability during voluntary muscle contractions. However, blockade of mAChRs revealed that cholinergic mechanisms affect inhibitory circuits associated with the SP during muscle contractions. These significant cholinergic effects were only apparent for unfatigued voluntary contractions, which may indicate that mAChR activity has a limited contribution to fatigue-related processes in the motor system.

## Data Availability

The datasets generated during and/or analysed during the current study are available from the corresponding author on reasonable request.
